# Novel insights into maladaptive behaviours in Prader–Willi syndrome: serendipitous findings from an open trial of vagus nerve stimulation

**DOI:** 10.1111/jir.12203

**Published:** 2015-05-27

**Authors:** K. E. Manning, C. J. McAllister, H. A. Ring, N. Finer, C. L. Kelly, K. P. Sylvester, P. C. Fletcher, N. W. Morrell, M. R. Garnett, M. R. A. Manford, A. J. Holland

**Affiliations:** ^1^ Department of PsychiatryUniversity of CambridgeCambridgeUK; ^2^ National Institute for Health Research (NIHR) Collaborations for Leadership in Applied Health Research and Care (CLAHRC) East of EnglandCambridgeUK; ^3^ Cambridgeshire and Peterborough NHS Foundation TrustCambridgeUK; ^4^ National Centre for Cardiovascular Prevention and OutcomesUCL Institute of Cardiovascular SciencesLondonUK; ^5^ Addenbrooke's HospitalCambridgeUK; ^6^ Department of MedicineUniversity of CambridgeCambridgeUK; ^7^ Papworth Hospital NHS TrustCambridgeUK; ^8^ Bedford Hospital NHS TrustBedfordUK

**Keywords:** hyperphagia, maladaptive behaviour, Prader–Willi syndrome, social functioning, vagus nerve stimulation

## Abstract

**Background:**

We report striking and unanticipated improvements in maladaptive behaviours in Prader–Willi syndrome (PWS) during a trial of vagus nerve stimulation (VNS) initially designed to investigate effects on the overeating behaviour. PWS is a genetically determined neurodevelopmental disorder associated with mild–moderate intellectual disability (ID) and social and behavioural difficulties, alongside a characteristic and severe hyperphagia.

**Methods:**

Three individuals with PWS underwent surgery to implant the VNS device. VNS was switched on 3 months post‐implantation, with an initial 0.25 mA output current incrementally increased to a maximum of 1.5 mA as tolerated by each individual. Participants were followed up monthly.

**Results:**

Vagal nerve stimulation in these individuals with PWS, within the stimulation parameters used here, was safe and acceptable. However, changes in eating behaviour were equivocal. Intriguingly, unanticipated, although consistent, beneficial effects were reported by two participants and their carers in maladaptive behaviour, temperament and social functioning. These improvements and associated effects on food‐seeking behaviour, but not weight, indicate that VNS may have potential as a novel treatment for such behaviours.

**Conclusions:**

We propose that these changes are mediated through afferent and efferent vagal projections and their effects on specific neural networks and functioning of the autonomic nervous system and provide new insights into the mechanisms that underpin what are serious and common problems affecting people with IDs more generally.

## Introduction

Prader–Willi syndrome (PWS) is a neurodevelopmental disorder arising from a lack of expression of maternally imprinted genes at chromosomal locus 15q11–13. PWS affects around 1 in 25 000–29 000 births (Whittington *et al*. 2001; Smith *et al*. 2003) and is associated with mild–moderate intellectual disability; low muscle tone; early failure to thrive; relative growth and sex hormone deficiencies; behavioural, social and psychiatric difficulties, including temper outbursts, need for routine, skin picking, repetitive and ritualistic behaviours, and poor social adjustment and severe overeating beginning in preschool years, which, if unchecked, is responsible for increased mortality (Holland *et al*. 2003; Schrander‐Stumpel *et al*. 2003; Stevenson *et al*. 2007).

This overeating appears to result from aberrant satiety signalling, with both behavioural and neuroimaging studies demonstrating that satiety responses are delayed and short‐lived in PWS but, crucially, are present and potentially malleable (e.g. Holland *et al*. 1993; Hinton *et al*. 2006). No effective treatments for overeating are presently available.

Vagus nerve stimulation therapy® (VNS; Cyberonics, TX, USA) is approved for the treatment of epilepsy and depression. A small device implanted in the upper chest stimulates the left vagus nerve via electrodes attached to the nerve in the neck. The vagus nerve is involved in satiety signals from the gastrointestinal system to the brain, and studies have reported weight loss in some patients undergoing VNS, as well as altered food cravings (Burneo *et al*. 2002; Bogacz & Asconape 2003; Roslin & Kurian 2003; Koren & Holmes 2006; Bodenlos *et al*. 2007; Pardo *et al*. 2007; Abubakr & Wambucq 2008; Kansagra *et al*. 2010), indicating potential utility of VNS for use in the obese population.

Although the overeating often severely limits independence, for many individuals with PWS and those who support them, once a system for controlling food intake has been established, some of the biggest daily difficulties arise from the wider maladaptive behaviours common to people with PWS. Around 70% of those with PWS are reported to show marked behavioural problems (Holland *et al*. 2003). Maladaptive behaviours may be exhibited in relation to food seeking; however, such behaviours extend beyond the food‐related problems to a more general difficulty with mood lability and changes to routines and expectations that can escalate from repetitive questioning to physically aggressive outbursts.

The initial aim of this study was to assess the safety, acceptability and efficacy of VNS as a novel treatment for hyperphagia in PWS. However, unexpected improvements in the rate and severity of maladaptive behaviours were spontaneously reported by two participants and their families (see succeeding text). Consequently, the study took on a new aim: that of investigating potential effects of VNS on maladaptive behaviours.

## Method

### Participants

Ethical review and approval was obtained from the Cambridgeshire 1 Ethics Committee. Three adults (one man and two women; aged 20, 23 and 28 years at implantation) with genetically confirmed PWS, body mass index > 30 and the capacity to consent were recruited to the study via the Prader–Willi Syndrome Association UK.

### Procedure

The VNS therapy system was surgically implanted under general anaesthetic beneath the left clavicle (Cyberonics model 102 generator), and electrodes were attached to the left vagus nerve (Cyberonics model 302 bipolar lead) according to standard practice (Landy *et al*. 1993).

Before VNS activation, participants attended three monthly overnight visits at the National Institute for Health Research/Wellcome Trust Clinical Research Facility, Cambridge, for baseline measurements of the initial outcome variables. VNS was switched on with the following initial stimulation settings: 0.25 mA output current, 30 Hz frequency, 500 μsec pulse width and a 30 s on‐time, 5 min off‐time cycle. Following activation, participants continued to attend monthly overnight visits at the Clinical Research Facility, where the VNS output current was increased in 0.25 mA steps to a maximum of 1.5 mA as tolerated by each individual, and outcome measures to assess safety, acceptability and efficacy were obtained. Visit frequency was reduced to bimonthly once established on the maximum output.

#### Measures of efficacy and mechanism

Weight and body composition were measured using air‐displacement plethysmography (BOD POD; Life Measurement, Inc., CA, USA). The quantity consumed during 1‐h free access to food with homogenous calorie content was recorded using a universal eating monitor (Sussex Ingestion Pattern Monitor, University of Sussex). The hospital anxiety and depression scale was administered to assess potential additional effects on mood (Zigmond & Snaith 1983).

#### Additional measures

Semi‐structured interviews were carried out with each participant and carer to characterise the unexpected spontaneous reports of marked, positive behavioural changes. These were analysed by an independent researcher using thematic analysis (Braun & Clarke 2008).

Daily observation records were also obtained for one participant from her residential college, covering the academic year prior to starting VNS and two terms of the academic year following VNS initiation. Records for the intervening academic year, during VNS activation and intensity increase, were unavailable. These narrative accounts were studied for evidence of behavioural changes following VNS using directed qualitative content analysis (Hsieh & Shannon 2005), with coding categories informed by the themes emerging from the interviews and determined by the data available. Mean occurrences per half term before and with VNS were calculated for each coded behaviour.

#### Measures of safety and acceptability

Owing to the reported association of VNS with increases in sleep apnoea (Marzec *et al*. 2003), sleep was monitored to ensure safety in respect to sleep apnoea using a Somnoscreen^TM^ plus RC system (SOMNOmedics, Germany) approximately every 3 months, with overnight oximetry at other visits. Heart rate and blood pressure were monitored during and for 4 h following VNS switch on and intensity increases, with pulse rate monitoring continuing throughout the night.

## Results

### Efficacy

#### Weight and body composition

Following VNS implantation, but prior to activation in January 2010, participant 1 started to gain weight, leading to cessation of VNS approximately 8 months after activation. VNS was reinstated 5 months later at the participant's request. Her weight remained elevated (at around 15–20 kg above baseline), but more stable. VNS was switched off at study conclusion in September 2012.

Participant 2 has had ongoing stimulation since February 2010, with no reported side effects. Her weight remained stable for the first 8 months of stimulation and, thereafter, showed a small and gradual decrease with some fluctuations. At study conclusion, his weight was 2.6 kg (2.9%) below his or her mean baseline weight of 90.4 kg, accounted for by loss of body fat alongside increased lean mass. Poor glycaemic control with energy loss from glucosuria may have contributed.

Participant 3 started VNS in February 2011. Her weight remained stable, with small fluctuations.

Data are available on request.

#### Eating behaviour

**Figure 1:**
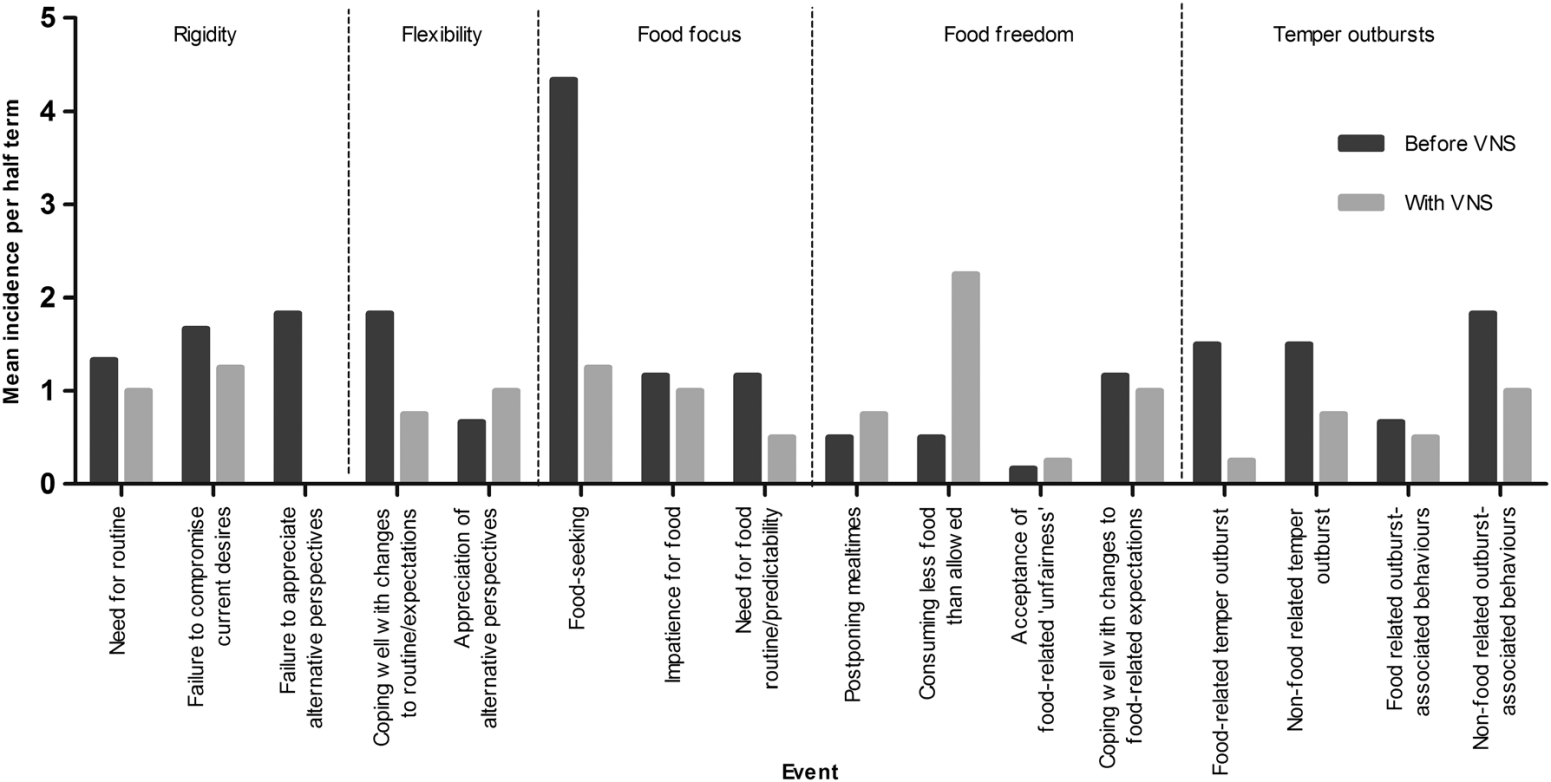
Mean incidence of behaviours per half term before and during vagal nerve stimulation (VNS) as reported in participant 3's daily observation records kept by the residential college at which he or she was a resident student.

Changes in everyday eating behaviour have been observed in two participants. Participants 2 and 3 and their respective carers reported occasions when they have been amenable to unexpected alterations to food routine, including missing meals. Such tolerance was never experienced prior to VNS. For participant 3, this has extended to voluntarily taking less than allowed at some mealtimes, corroborated by college records indicating an increase in instances of leaving food offered or taking less than allowed (Fig. 1). Participant 3 also reported decreased attempts to obtain or conceal extra food, supported by a reduction of reports of food‐seeking behaviour in her college records (Fig. 1). However, no participant showed a consistent reduction in quantity consumed during 1‐h *ad libitum* access to food under experimental conditions (data not shown).

#### Unexpected observations of behaviour and temperament change

Six organising themes emerged from semi‐structured interviews carried out to explore reports of positive effects on temperament, behaviour and social functioning: ‘unpredictability and cognitive inflexibility’, ‘volatile social relationships’, ‘controlling power of food’, ‘controlled mood and flexibility of thought’, ‘increased ability to interact socially’ and ‘freedom from food focus’ (Table 1). Two participants and two carers reported improvements in the behaviour and temperament of the individual with PWS. These improvements were demonstrated through changes in the three superordinate themes, ‘reactivity’, ‘social functioning’ and ‘control over food’, into which the six organising themes could be grouped, with each superordinate theme comprising one organising theme relating to life before VNS and another to life with VNS (Table 1). Overall, increased emotional and cognitive flexibility was described, enabling better handling of situations that would previously have caused a temper outburst, including changes to routines and expectations, with beneficial consequences for family life and social functioning.

**Table 1:** Thematic analysis of semi‐structured interviews exploring reported behavioural improvements

VNS status	Superordinate theme	Organising theme	Code
Before VNS	Reactivity	Unpredictability and cognitive inflexibility	Mood lability; inflexibility; frequent temper outbursts
	Social functioning	Volatile social relationships	Difficulty in social situations; family dysfunction
	Control over food	Controlling power of food	Ruled by food; stressful impact on carer's daily life/routine
With VNS	Reactivity	Controlled mood and flexibility of thought	Infrequent temper outbursts; calm/happier mood; low anxiety; increased flexibility of thought/ability to reason; sense of well‐being
	Social functioning	Increased ability to interact socially	Coping in social situations; enhanced family functioning
	Control over food	Freedom from food focus	Liberating effect of VNS; lower focus on food; slight lessening of hunger; improved carer's mood

VNS, vagal nerve stimulation.

No such changes were reported by participant 1 and her carer. However, participant 1 did not experience the same level of behavioural difficulties prior to VNS.

Analysis of participant 3's college records suggested reduced rigidity, better appreciation of alternative perspectives, reduced food focus (especially food seeking), generally increased food freedom (particularly consuming less than allowed) and reduced temper outbursts and outburst‐associated behaviours with VNS (Fig. 1). Only two sub‐codes did not fit this pattern of improvement and were related to instances of coping well with expectation change. While these appear to question improvement in this area, negative instances of this behaviour (i.e. demonstrating need for routine) were reduced.

For all participants, hospital anxiety and depression scale scores remained low throughout the study (data not shown), suggesting improvements could not be simply be explained by the alleviation of previously undiagnosed depressive mood.

### Safety and acceptability

Participant self‐reports suggested few side effects of VNS; two participants experienced voice hoarseness during stimulation, but this was well tolerated.

Few safety concerns arose. An increase in obstructive events in participant 1's pre‐existing obstructive sleep apnoea resolved with alteration of established continuous positive airway pressure settings. This increase corresponded to participant 1's weight gain, suggesting that it was not directly VNS related.

Participant 3 reported dyspnoea during physical exertion following VNS output reaching 1.0 mA. VNS was temporarily ceased, but comprehensive tests of lung function found no detrimental effects of VNS on respiration, and stimulation was reinstated 1 month later with no re‐emergence of symptoms. This is a commonly reported side effect of VNS and is not thought to present any danger (Handforth *et al*. 1998).

## Discussion

The initial aims of this study were to investigate the safety and acceptability of VNS in adults with PWS and its possible efficacy in reducing hyperphagia. Other than tolerable effects on voice, no side effects or significant safety issues were found. VNS had no direct effect on reducing food intake, and only one participant showed minimal weight loss. There were, however, unexpected clinically significant emotional and behavioural benefits of VNS in the two participants who had previously shown significant problems in these areas. Improvements in flexibility around food routine and food‐seeking behaviour were associated with reduced temper outbursts and difficult behaviour when mealtimes were unexpectedly postponed or a desired food was denied. Furthermore, these changes extended beyond food‐related events to other situations that would previously have resulted in difficult behaviour.

The observed effects were sufficient to lead to spontaneous and independent reports by family and to the request for continuation of VNS therapy despite the absence of significant weight loss. As these behavioural effects were not anticipated, quantitative prestimulation behavioural data were unavailable. However, the reported changes were largely corroborated by participant 3's college records. Despite clear limitations, these documents provide a valuable record of behaviour both prior to and during a period of VNS, written contemporaneously by carers independent of the research study.

Conclusions are also limited by the small number of participants and non‐random selection – a result of the invasive nature of the intervention and the uncertainty of outcome – and the lack of prospective measures to assess these serendipitous findings. Furthermore, blinding was not possible, owing to the need to ensure participant safety during this novel treatment in PWS, as well as noticeable effects on the voice during stimulation. However, it appears unlikely that improvements arose because of non‐treatment study‐related factors, such as expectation, attention and feedback, given that the reported improvements were largely unanticipated in nature and intensive monitoring had been in place for a number of months prior to VNS activation without such changes occurring. Moreover, such factors did not enable a reduction in food intake during even a short hour‐long food test.

Effects on behaviour and social and cognitive functioning have previously been identified in the VNS literatures potentially independent from the effects of VNS on seizure control (e.g. Rawlins 1997; Galli *et al*. 2003; Kossoff & Pyzik 2004; Cukiert *et al*. 2009; Alonso‐Vanegas *et al*. 2010; Achinivu *et al*. 2012). Importantly, the current study provides evidence for improvements in global functioning with VNS in individuals whose psychobehavioural symptoms are not secondary to depression or seizures. This suggests that behavioural benefits of VNS can be independent from its anticonvulsant and antidepressant effects.

We propose that these incidental observations indicate the possibility that the predisposition to such maladaptive behaviours has a biological basis mediated on the basis of the influence of afferent and efferent vagal projections on specific neural networks and functioning of the autonomic nervous system (in line with Porges's polyvagal theory; Porges & Furman 2011).

## Source of funding

This study was funded by The Dunhill Medical Trust, Addenbrooke's Charitable Trust, Isaac Newton Trust and Prader–Willi Association UK. Funding bodies had no role in the study design, data collection, data analysis, data interpretation, writing of the report or the decision to submit for publication. We are grateful to the NIHR Collaborations for Leadership in Applied Health Care Research and Care (CLAHRC) East of England for the financial support to AJH and HAR and to the Health Foundation for the support of AJH. The views expressed are those of the authors and not necessarily those of the NHS, the NIHR or the Department of Health.

## Conflict of Interest

The authors report no conflicts of interest. The authors alone are responsible for the content and writing of the paper.
